# Poly(Arylene Alkylene)s with Tetrazole Pendants for Alkaline Ion‐Solvating Polymer Electrolytes

**DOI:** 10.1002/cssc.202400844

**Published:** 2024-08-08

**Authors:** Yifan Xia, Sinu C. Rajappan, Si Chen, Mikkel Rykær Kraglund, Dmytro Serhiichuk, Dong Pan, Jens Oluf Jensen, Patric Jannasch, David Aili

**Affiliations:** ^1^ Department of Energy Conversion and Storage Technical University of Denmark Elektrovej, Building 375 Lyngby 2800 Denmark; ^2^ Polymer & Materials Chemistry Department of Chemistry Lund University P.O. Box 124 22100 Lund Sweden

**Keywords:** Ion-solvating membrane, Alkaline water electrolysis, Polyhydroxyalkylation, Tetrazole functionalization, Conductivity

## Abstract

Alkaline ion‐solvating membranes derived from a tetrazole functionalized poly(arylene alkylene) are prepared, characterized and evaluated as electrode separators in alkaline water electrolysis. The base polymer, poly[[1,1′‐biphenyl]‐4,4′‐diyl(1,1,1‐trifluoropropan‐2‐yl)], is synthesized by superacid catalyzed polyhydroxyalkylation and subsequently functionalized with tetrazole pendants. After equilibration in aqueous KOH, the relatively acidic tetrazole pendants are deprotonated to form the corresponding potassium tetrazolides. The room temperature ion conductivity is found to peak at 19 mS cm^−1^ in 5 wt. % KOH, and slightly declines with increasing KOH concentration to 13 mS cm^−1^ in 30 wt. % KOH. Based on an overall assessment of the mechanical properties, conductivity and electrode activity, 30 wt. % KOH is applied for alkaline electrolysis cell tests. Current densities of up to 1000 mA cm^−2^ were reached with uncatalyzed Ni‐foam electrodes at a cell voltage of less than 2.6 V, with improved gas barrier characteristics compared to that of the several times thicker Zirfon separator.

## Graphical abstract

A poly(arylene alkylene) equipped with tetrazole pendants is synthesized via polyhydroxyalkylation and evaluated as ion‐solvating polymer electrolyte for alkaline water electrolysis. The electrolyte uptake and conductivity can be tuned by adjusting the KOH concentration, and the technological feasibility is demonstrated in electrolysis cell tests with nickel foam electrodes in 30 % KOH.

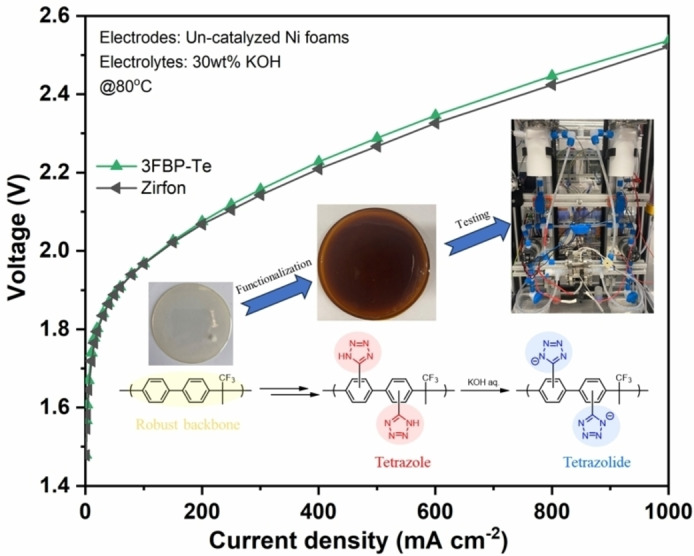

## 1 Introduction

Alkaline ion‐solvating membranes doped with aqueous KOH is a promising class of electrode separators for high‐rate alkaline water electrolysis, as they combine high ion conductivity with low gas crossover.^[1]^ Like porous diaphragms, the ion conductivity is supported by the aqueous electrolyte within the materials matrix, but in contrast to the diaphragms the electrolyte is accommodated in a swollen polymer matrix instead of in distinct micrometer sized pores.^[2]^ The effective pore dimensions are therefore defined by the polymer chain separation, which is in the range of 10–20 nm for membranes based on poly(2,2′‐(*m*‐phenylene)‐5,5,–bibenzimidazole) (*m*‐PBI) prepared by solution casting from organic solvents.^[3]^

The electrolyte uptake of *m*‐PBI depends strongly on the KOH concentration of the surrounding solution, which also determines the fundamental physicochemical characteristics of the membrane.[^4^, ^5^, ^6^] The conductivity at 80 °C can reach as high as 100–200 mS cm^−1^ in 15–25 wt. % KOH,^[7]^ which allows for remarkable performance characteristics when combined with highly active electrodes^[8]^ and lifetimes exceeding 1000 h when mechanically reinforced.^[9]^ The remaining challenge is to mitigate degradation of the polymer backbone, which results in gradual decay of the molecular weight and eventually membrane failure.^[10]^ In line with the successful design principles of alkaline resilient dialkyl poly(benzimidazoliums),[^11^, ^12^] alternative polybenzimidazole chemistries with steric groups installed in the vicinity of the labile linkages[^13^, ^14^, ^15^] and reduced KOH concentrations^[16]^ have been explored for doping with aqueous KOH.

Recent studies indicate that the backbone degradation of polybenzimidazoles primarily proceeds by an initial nucleophilic attack at the C2 position of the fraction of neutral (non‐deprotonated) benzimidazole groups.[^17^, ^18^] These findings could potentially reveal new design strategies to improve the chemical stability, but the stability challenges have also stimulated the development of alternative membrane chemistries based on e. g. poly(arylene ether sulfone) blends with poly(vinyl pyrrolidone),^[19]^ poly(vinyl alcohol),^[20]^ styrene‐ethylene‐butylene copolymers,^[21]^ imidazole functionalized poly(arylene alkylene)s,^[22]^ as well as polyisatin^[23]^ and its blends with *m*‐PBI.[^24^, ^25^]

In response to the backbone instability of common arylene‐ether containing polymers for anion exchange membrane applications, poly(arylene alkylene)s have been intensively explored since they were first reported by Lee et al.^[26]^ and the synthetic methodology has paved the way for new structures based on e. g. poly(arylene piperidinium)s,[^27^, ^28^, ^29^] poly(terphenyl alkylene)s^[30]^ and polycarbazoles.^[31]^

Tetrazole has recently been identified as a remarkably stable structure element in alkaline ion solvating membranes,^[32]^ and this work describes the installation of tetrazoles onto poly(arylene alkylene)s based on poly[[1,1′‐biphenyl]‐4,4′‐diyl(1,1,1‐trifluoropropan‐2‐yl)] (3FBP). The tetrazole‐grafted 3FBP was accessed through superacid catalyzed polyhydroxyalkylation,[^32^, ^33^] and the tetrazole functionalities were introduced by bromination of the 3FBP, conversion to the corresponding nitrile, followed by a click‐type cycloaddition. After casting to homogenous films, the membranes were extensively characterized with respect to electrolyte uptake, swelling behavior and conductivity and evaluated in alkaline electrolysis tests, showing comparable polarization performance and better gas resistance than that of state‐of‐the‐art separators.

## 2 Results and Discussion

### 2.1 Synthesis, Membrane Preparation and Characterization

**Figure 1:**
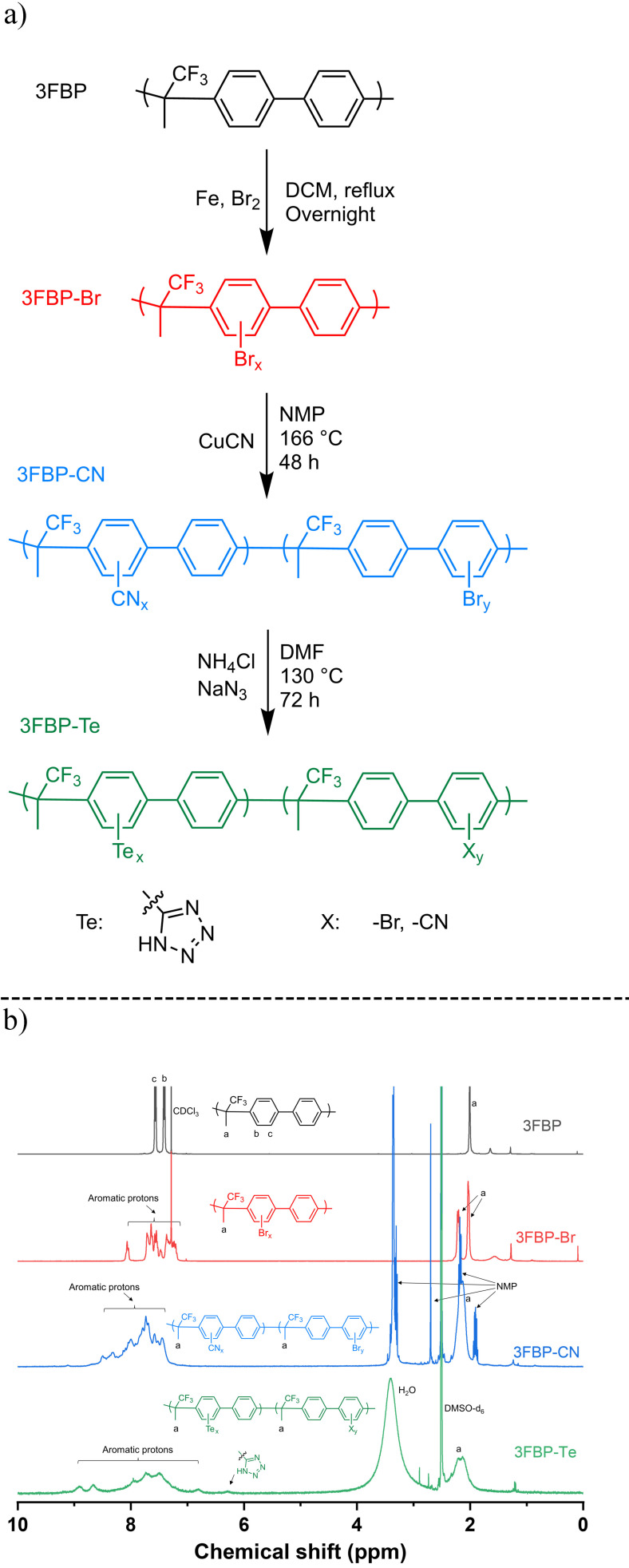
a) Synthesis of 3FBP‐Te from 3FBP via bromination, nitrile substitution and [3+2] cycloaddition. b) ^1^H NMR spectra of 3FBP, 3FBP‐Br, 3FBP‐CN, and 3FBP‐Te.

The base polymer 3FBP shown in Figure 1a was synthesized by a superacid‐catalyzed polyhydroxyalkylation reaction between a trifluoromethyl‐activated ketone and biphenyl and driven by the elimination of water, in line with the methodology described by Guzmán‐Gutiérrez et al.^[35]^ To avoid gelation during the polymerization, the monomer concentration and the mole ratio between TFSA and the ketone monomer was kept low. This produced a well‐defined polymer with an inherent viscosity *η*_inh_ of 0.83 dL g^−1^ and *M*_w_ of 10.9×10^4^ g mol^−1^ (PDI=1.28), as determined by SEC. The ^1^H NMR spectrum of 3FBP and peak assignments are shown in Figure 1b, and the other functionalized 3FBP samples with the most probable structures are also presented, with clear peak at 7.4 and 7.6 ppm corresponding the arylene protons and a peak at 2.0 ppm corresponding to the methyl group of the alkylene linkage.

The obtained 3FBP combined good solubility and prcessability with excellent film‐forming properties, and was brought forward for bromination by electrophilic substitution in DCM with molecular bromine and ferric bromide as catalyst.^[36]^ After the random bromination, a new peak at around 8.1 ppm was observed, which is related to the proton adjacent to the introduced bromine atoms of the 3FBP‐Br.

The subsequent substitution of the aryl bromides with aryl nitriles was accomplished via the Rosenmund‐von Braun reaction, by reacting 3FBP‐Br with CuCN in NMP.^[37]^ The obtained aryl nitrile functionalized polymer 3FBP‐CN showed a poorly resolved aromatic region in the ^1^H NMR due to the limited solubility of 3FBP‐CN and the complex conjugation involving the nitrile groups.^[38]^

The final synthetic step to convert the aryl nitrile groups to the corresponding tetrazoles was carried out by a [3+2] cycloaddition click reaction between the aryl nitrile and an azide salt.^[39]^ The ^1^H NMR spectrum of the obtained 3FBP‐Te sample showed a shift to lower field for the arylene protons, due to the introduction of the strongly electron withdrawing nitrile groups. A new peak appeared at around 6.23 ppm, corresponding to the tetrazole proton.

After isolation and work‐up of 3FBP‐Te it was dissolved in NMP and cast on glass by solvent evaporation to form mechanically robust and homogenous films, as shown in Figure S1.

**Figure 2:**
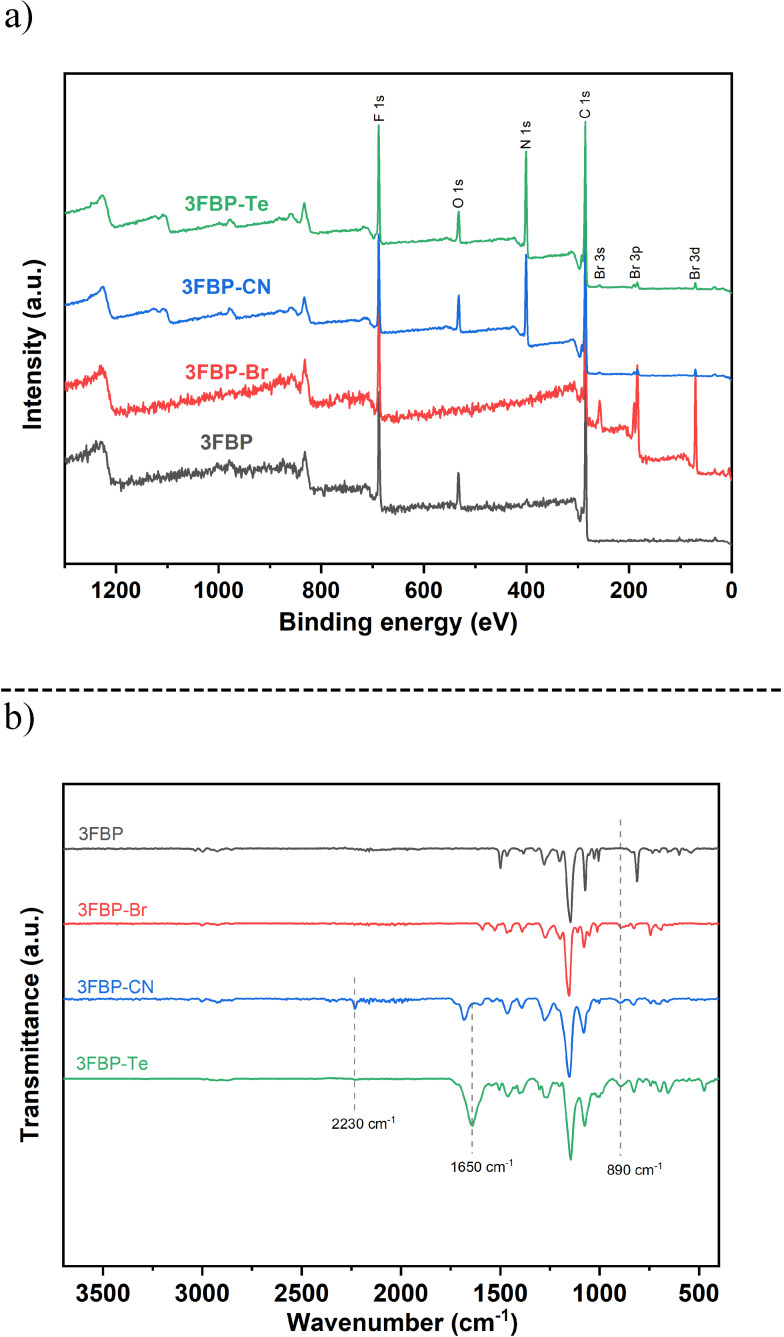
Survey scan XPS (a) and ATR‐FTIR spectra (b) for 3FBP, 3FBP‐Br, 3FBP‐CN and 3FBP‐Te.

As discussed above, the initial bromination occurred randomly and dictated the functionalization pattern of the subsequent steps. Due to a significant signal overlap and poorly resolved signals in the ^1^H NMR spectrum, the degree of functionalization was instead determined by XPS. Figure 2a shows the wide scan XPS for 3FBP, 3FBP‐Br, 3FBP‐CN and 3FBP‐Te. For the pristine 3FBP membrane, only fluorine and carbon could be observed. However, oxygen was also detected, likely due to the presence of water within the membrane. Similar water absorption was found for 3FBP‐CN and 3FBP‐Te. For 3FBP‐Br, new peaks were detected at 256, 187 and 72 eV, corresponding to Br 3s, Br 3p and Br 3d respectively.^[40]^

In order to determine the bromination degree of the 3FBP‐Br, the three fluorine atoms in the structural repeat unit was used as internal standard for normalization. The fractional concentration of the different elements is presented in Table 1, where the bromination degree was calculated to be 166 %. The compositions were calculated from the peak deconvoluted spectra as shown in Figure S2 and S3. The deconvoluted C1s result of 3FBP‐Br in Figure S2 shows a new peak around 268.1 eV, which is due to the presence of C−Br bonds.[^40^, ^41^] In addition, only one set of spin orbit doublets from Br 3d deconvoluted spectrum was found, which implies that the free and ionic bromide residuals had been washed away thoroughly.[^41^, ^42^] For 3FBP‐CN, a new peak around 400 eV was observed, due to the introduction of nitrile groups. The deconvoluted results for C1s spectrum of 3FBP‐CN exhibited a new peak around 287.5 eV, which is attributed to the binding energy of the C=N bond within the nitrile group.[^42^, ^43^, ^44^] The content of nitrile groups in 3FBP‐CN was calculated to be 150 %, which indicates a 91 % conversion ratio from bromine to nitrile. Moreover, it was found that 16 % of the bromine had not been converted, which translates to 166 % total functional degree for 3FBP‐CN. This result is in good agreement with the initial bromination degree of 3FBP‐Br. After the [3+2] cycloaddition click reaction, the content of nitrogen in the polymer was found to increase dramatically and the degree of functionalization of 3FBP‐Te was calculated to be 158 %. This corresponds to >100 % conversion, which is due to complete conversion of the nitrile functions to tetrazole and substitution of bromine residuals with azide as NaN_3_ was used in excess.

**Table 1:** Composition and the group content of 3FBP, 3FBP‐Br, 3FBP‐CN and 3FBP‐Te obtained from XPS. Br, CN and Tetrazole content is normalized to 3 fluorine atoms representing one repeat unit.

Membrane	C1s	N1s	F1s	Br3d	[Br]/[F]	[N]/[F]	Br content	CN content	Tez content
							3[Br]/[F]	3[N]/[F]	3[N]/4[F]
	(atom %/ ratio %)								
3FBP	87.4	–	12.6	–	–	–	–	–	–
3FBP‐Br	74.1	–	16.7	9.2	55.3	–	165.9	–	–
3FBP‐CN	84.7	4.9	9.9	0.5	5.4	50	16.1	150.2	–
3FBP‐Te	73.0	18.1	8.6	0.3	2.9	211	8.7	–	158.1

From the FTIR results in Figure 2b, a new absorption band around 890 cm^−1^ could be observed after the initial bromination step, which is due to the random functionalization of the aromatic rings.^[45]^ The typical absorption band for nitrile can be seen at around 2230 cm^−1^. For 3FBP‐Te, the FTIR spectrum reveals the disappearance of nitrile, and instead shows the typical tetrazole ring stretching vibration around 1650 cm^−1^.[^46^, ^47^]

**Figure 3:**
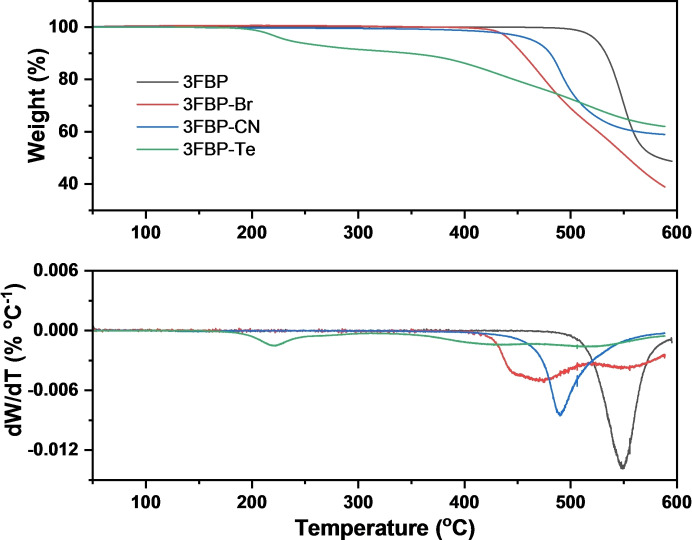
Thermogravimetric curves (top) and derivative of weight loss (bottom) of 3FBP, 3FBP‐Br, 3FBP‐CN and 3FBP‐Te, respectively.

The thermogravimetric curves and corresponding derivative of the weight loss of 3FBP, 3FBP‐Br, 3FBP‐CN and 3FBP‐Te are shown in Figure 3. For 3FBP, the weight remained constant until the temperature reached around 500 °C, corresponding to degradation of the backbone. The onset of major decomposition of 3FBP‐Br and 3FBP‐CN was around 420 and 430 °C, respectively. For the 3FBP‐Te, a major degradation step started at around 200 °C, which could be attributed to opening of the tetrazole rings.[^48^, ^49^] The subsequent weight losses at 380 and 480 °C are related to the decomposition of the opened tetrazole residuals and backbone, respectively.

**Figure 4:**
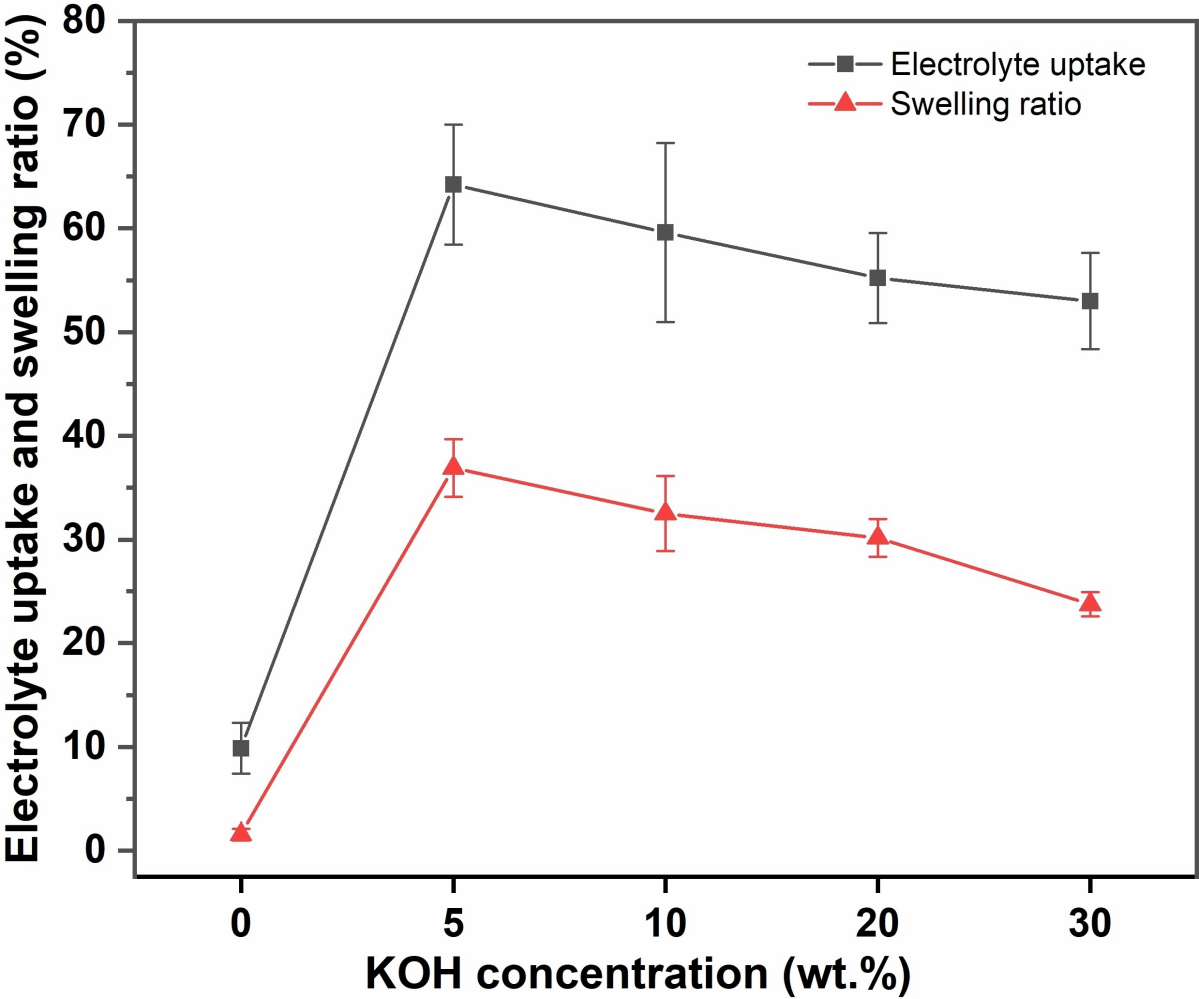
Electrolyte uptake and swelling of 3FBP‐Te as recorded at room temperature in aqueous KOH with concentrations of 0 (pure water), 5, 10, 20 and 30 w %. The markers show the average of three samples, and the error bars represent the standard deviation.

The electrolyte uptake and swelling of the 3FBP‐Te membrane in 0–30 wt. % KOH is presented in Figure 4. The electrolyte uptake was found to peak at 64 wt. % in 5 wt. % KOH, and thereafter declined with increasing KOH concentration. For comparison, the electrolyte uptake of *m*‐PBI peaks at around 25 wt. % KOH.^[7]^ The discrepancy is likely due to the significant difference in acidity between benzimidazole and tetrazole, with p*K*_a_ values of around is 12.8 and 4.9 respectively.[^50^, ^51^] This implies that the 3FBP‐Te is readily deprotonated even at relatively low KOH concentrations to form negatively charged potassium tetrazolide pendants along the backbone. This increases the polarity of the polymer, which could contribute to an increased electrolyte uptake, but may be counterbalanced by the electrostatic repulsion of hydroxide ions due to Donnan exclusion. The situation may be similar to a perfluorosulfonate membrane equilibrated in KOH, which shows decreasing electrolyte uptake with increasing KOH concentration.[^52^, ^53^] Decreasing electrolyte uptake with increasing KOH concentration has also been observed for anion exchange membranes derived from *m*‐PBI.^[54]^ The through‐plane swelling ratio of the 3FBP‐Te membrane followed the same trend as the gravimetric electrolyte uptake, with a peak swelling of 37 % in 5 wt. % KOH.

The surface and cross‐sectional SEM images of the 3FBP‐Te membrane, shown in Figure S4, did not present any morphological features such as porosity or phase separation at the micrometer length scale. The random functionalization of the polymer chain results in a homogenous phase throughout the membrane. The thickness normalized XRD patterns of 3FBP, 3FBP‐Br, 3FBP‐CN and 3FBP‐Te after casting from NMP are shown in Figure S5a. For 3FBP, it shows two close broad peaks around 2*θ*=12.8° and 16.1°, which indicates that 3FBP has two different orientations of amorphous phase with *d*‐spacing of 6.9 and 5.5 Å, respectively. However, when it comes to 3FBP‐Br and 3FBP‐CN, the diffraction intensity was found to decrease. This may be a result of the initial random bromination, resulting in a more amorphous structure. The diffraction peaks were also shifted to higher 2*θ* angles, which indicates smaller *d*‐spacing. This could be due to the stronger interaction between the polar bromine and nitrile groups along the backbone, and the extensive hydrogen bonding network for 3FBP‐Te resulting in close chain packing from the tetrazole rings.^[6]^

After equilibration in aqueous KOH, the 3FBP‐Te membrane exhibited lower amorphous scattering intensity compared to the dry membrane, as shown in Figure S5b. This is likely due to the polymer chain separation resulting from the electrolyte uptake. When the concentration of the doping electrolytes increased from 5 to 20 wt. %, the amorphous intensity of doped membranes also increased. This might be due to the lower electrolyte uptake resulting in close polymer chain stacking. However, comparing 20 wt. % with 30 wt. % KOH doped membrane, they present almost the same amorphous intensity, which is likely due to their similar electrolytes uptake. It could also be observed there are several new sharp peaks for 30 wt. % KOH doped 3FBP‐Te, which is possibly due to the presence of free KOH within the membrane.[^55^, ^56^] However, it is known that the dry or hydrated KOH could present more complex diffraction patterns. These only four sharp peaks might be from the crystalline phase of the formed polymer potassium tetrazolides salt, as previously observed for *m*‐PBI membrane doped with highly concentrated KOH.^[6]^

**Figure 5:**
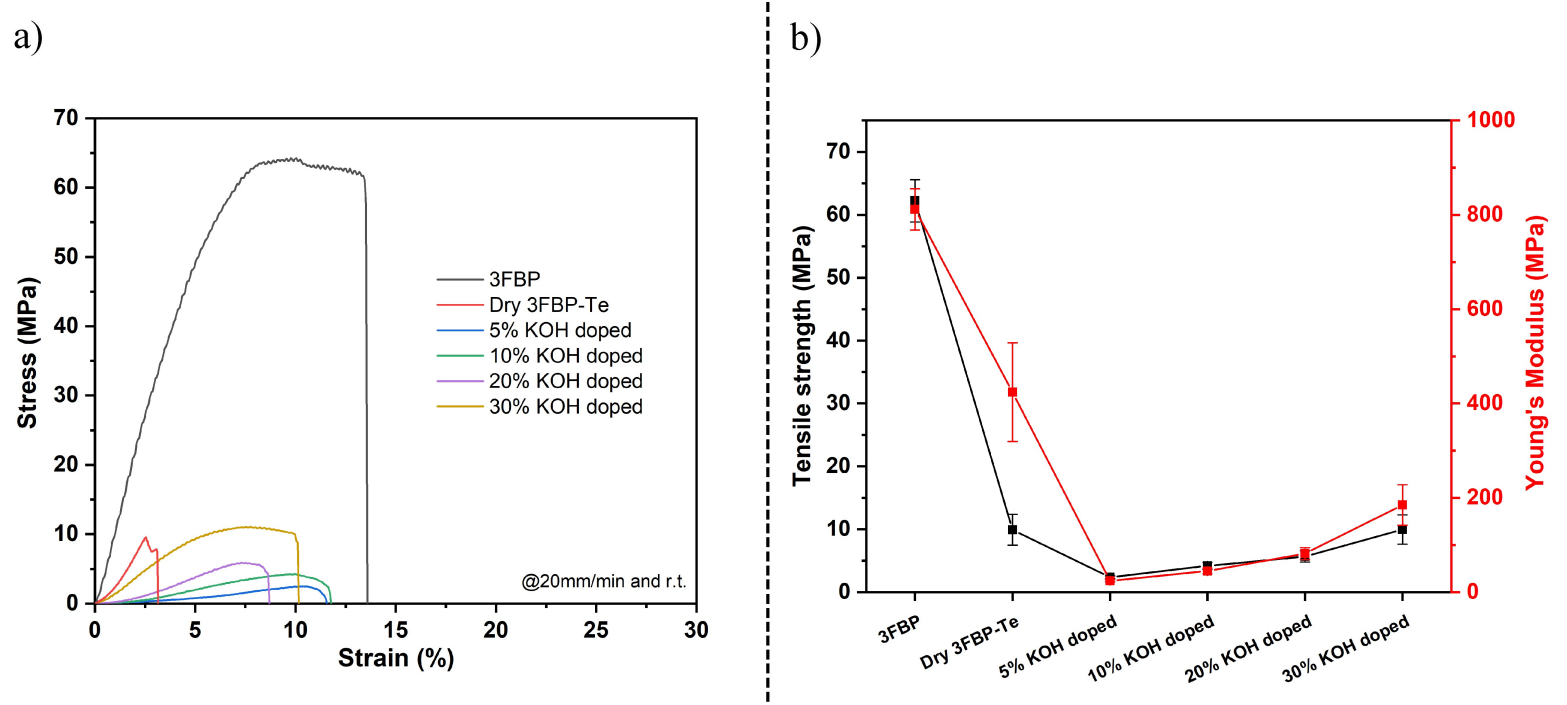
Representative stress‐strain curves of pristine 3FBP and 3FBP‐Te before and after equilibration in aqueous KOH with concentrations of 5–30 wt. % (a), and corresponding tensile strength and Young′s modulus (b). The markers show the average of at least three samples, and the error bars represent the standard deviation.

Figure 5 shows representative stress‐strain curves for pristine 3FBP, as well as 3FBP‐Te, before and after equilibration in aqueous KOH with concentration of 5–30 wt. %. It can be seen that the introduction of tetrazole pendants on the 3FBP backbone had a major impact on the mechanical characteristics at the membrane level. Even though the additional hydrogen bond donor/acceptors potentially could govern intermolecular interaction of the polymer chains, the plasticizing effect by the pendants were dominating. After equilibration in aqueous KOH, the membrane showed a higher degree of plastic deformation. This is a result from the ion‐solvation process between KOH and tetrazole, which results in increasing plasticization. The minima in tensile strength and elastic modulus of 2.3 and 24.1 MPa, respectively, were found to coincide with the peak in electrolyte uptake, which is in line with the mechanical response of *m*‐PBI membranes in aqueous KOH.^[6]^

### 2.2 Conductivity and Water Electrolysis Tests

**Figure 6:**
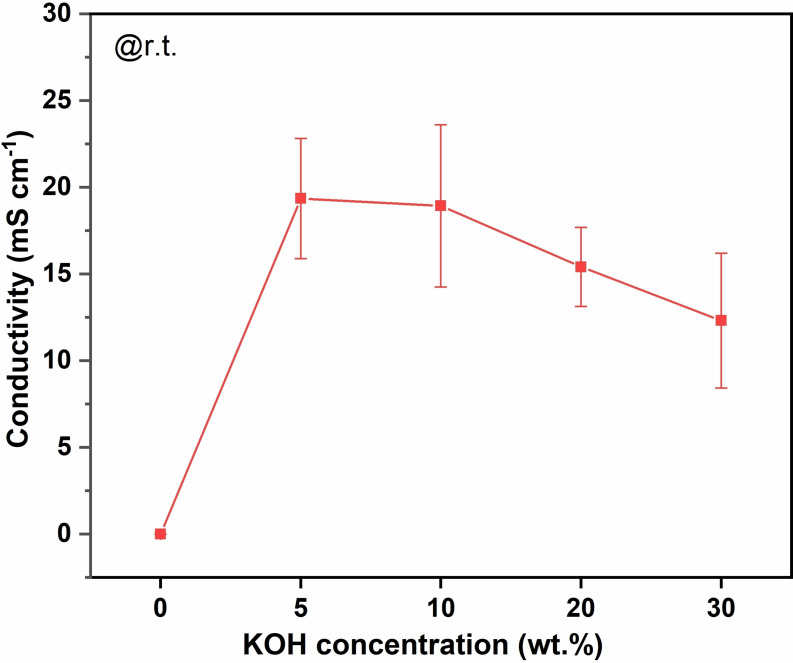
Room temperature through‐plane ion conductivity of 3FBP‐Te in 0–30 wt. % KOH. The markers show the average of three samples, and the error bars represent the standard deviation.

At room temperature, the through‐plane ion conductivity of 3FBP‐Te was found to peak at 19 mS cm^−1^ in 5 wt. % KOH, and thereafter decreased slightly to 13 mS cm^−1^ when the concentration was increased to 30 wt. % KOH, as shown Figure 6. For comparison, the room temperature conductivity of *m*‐PBI in 5 wt. % KOH is <1 mS cm^−1^, although it increases to around 100 mS cm^−1^ when the KOH concentration is increased to 20–25 wt. %.^[7]^

From a conductivity point of view, 5 wt. % KOH appears to be the optimal operating point for the electrolysis tests. However, the cell tests were conducted in 30 wt. % KOH to enhance the mechanical robustness of the membrane and to reduce the chance of premature failure due to excessive swelling. The higher operating concentration was also expected to improve electrode kinetics, bulk electrolyte ionic transport within the porous foam electrodes, and to reduce hydrogen crossover compared with lower KOH concentration feeds,^[1]^ and facilitate the comparison with the conventional porous electrode separator (Zirfon). An overview of the 3FBP‐Te characteristics in 30 wt. % KOH is shown in Table 2.

**Table 2:** Overview of key characteristics of the 3FBP‐Te in 30 wt. % KOH at room temperature.

Membrane	Thickness (wet)	Tensile strength (wet)	Young′s Modulus (wet)	Area specific resistance	Conductivity
	(μm)	(MPa)	(MPa)	(Ω cm^2^)	(mS cm^−1^)
3FBP‐Te	95	10	184	0.73	13
Zirfon	500	–	–	0.39	128

For cell testing, the 3FBP‐Te membranes were sandwiched between a pair of uncatalyzed Ni foam electrodes and mounted in a 10 cm^2^ active area single cell. Initial polarization curves for 3FBP‐Te and Zirfon were obtained at 40, 60 and 80 °C as shown in Figure S6a, S6b and S6c, respectively.

**Figure 7:**
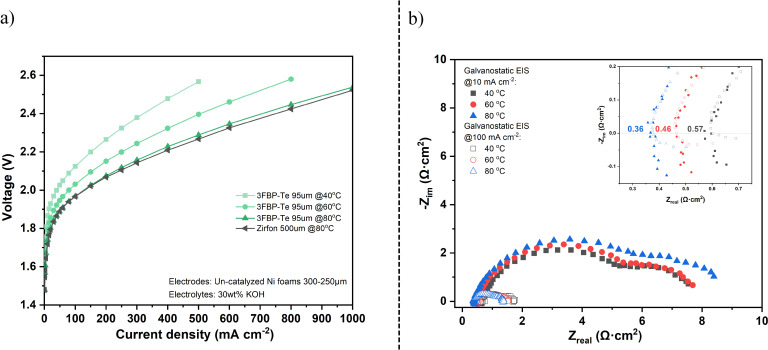
Water electrolysis polarization curves the cell equipped with 3FBP‐Te at 40–80 °C (a) along with corresponding galvanostatic EIS (b).

The polarization data for 3FBP‐Te at 40–80 °C are compared in Figure 7a, and the corresponding galvanostatic and potentiostatic EIS are shown in Figure 7b and S7, respectively. The overpotentials in the lower current density range are mainly originating from the faradaic processes at the electrodes, while the ohmic resistance of the membrane dominates in the linear part of the curves. With increasing temperature, the polarization performance of the cell assembled with the 3FBP‐Te membrane showed a gradual improvement, and was found to perform similar to Zirfon at 80 °C. This may be due to an increasing electrolyte uptake of 3FBP‐Te with increasing temperature, which leads to a higher conductivity increase than what is observed for Zirfon.

The ASR obtained from the high frequency intersection of the galvanostatic (at 10 and 100 mA cm^−2^) and potentiostatic (at 1.3 V) EIS data in Figure 7b and S7, respectively, was found to correspond to an in‐situ conductivity of 17, 21 and 26 mS cm^−1^ at 40, 60 and 80 °C, respectively. However, the specific conductivity calculated based on the resistance obtained from the linear regression of the polarization curves in the 200–1000 mA cm^−2^ were apparently lower, as a result of porous electrode behavior.^[57]^ At 40, 60 and 80 °C, it was estimated to be 12, 13 and 17 mS cm^−1^, respectively. While the potentiostatic EIS was recorded at 1.3 V, where no gas evolution occurs, the galvanotatic EIS were recorded at 10 and 100 mA cm^−2^. In this case, entrapment of evolved gases could lead to increased resistance, which is likely the main reason for the discrepancy between the potentiostatic and galvanostatic EIS data.

**Figure 8:**
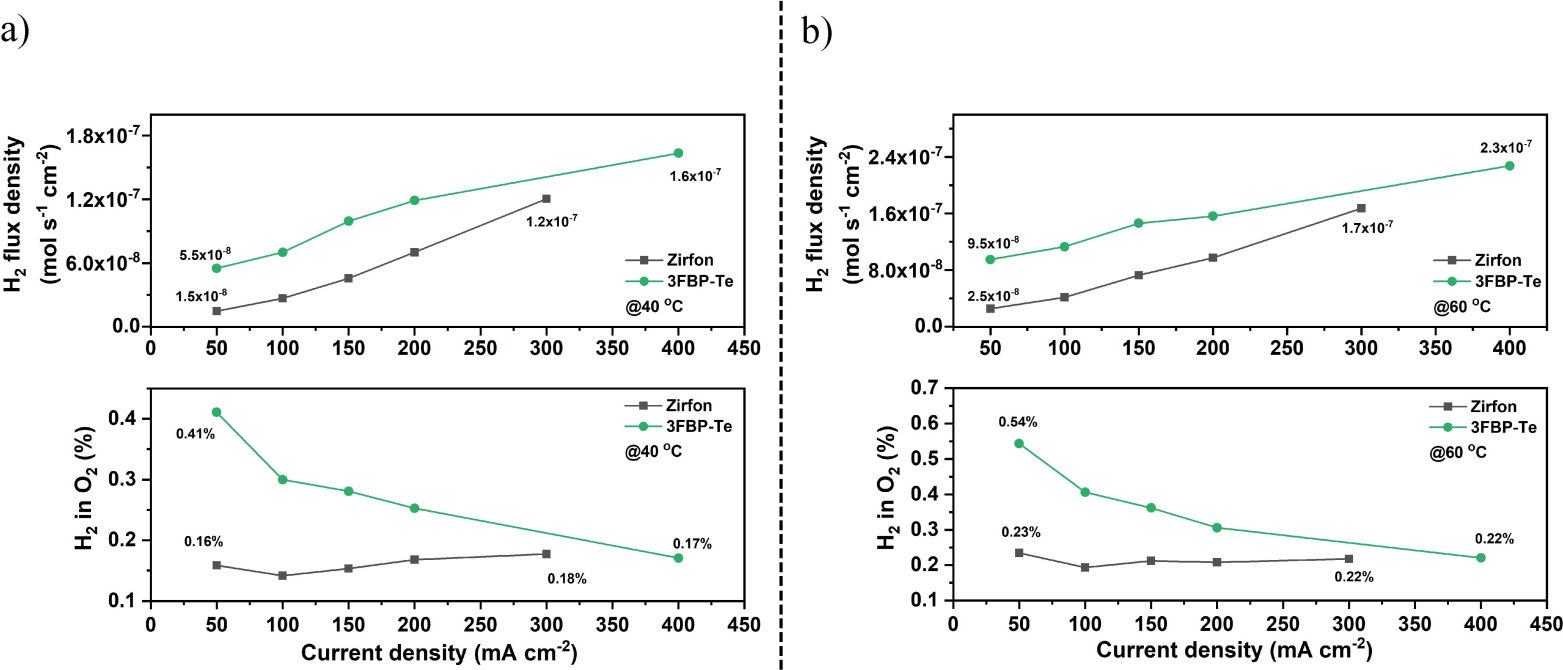
Hydrogen flux density and H_2_ in O_2_ levels at 40 °C (a) and 60 °C (b) during water electrolysis tests in 30 wt. % KOH for cells equipped with 3FBP‐Te and Zirfon.

The cell voltage and hydrogen crossover evolution of a cell operated at 40 and 60 °C is shown in Figure S8. The H_2_ flux density and the corresponding H_2_ in O_2_ level (HTO) at 40 and 60 °C are shown in Figure 8a and 8b, respectively. It can be seen that the H_2_ in O_2_ level at the anode decreased with increasing current density for 3FBP‐Te as expected due to the increased dilution by the generated O_2_.^[58]^ For the H_2_ flux density, both of the cells assembled with 3FBP‐Te and Zirfon showed increasing values with increasing current density within the same order of magnitude, even though 3FBP‐Te was much thinner. At 40 °C and at 50 mA cm^−2^, the H_2_ crossover of 3FBP‐Te corresponded to a specific apparent permeability of 5.2×10^−10^ mol s^−1^ cm^−1^ bar^−1^, which is similar to that of Zirfon (7.4×10^−10^ mol s^−1^ cm^−1^ bar^−1^). However, with increasing current density and temperature, the apparent H_2_ permeability 3FBP‐Te was found to increase slower than for Zirfon. Especially, the cell assembled with 3FBP‐Te exhibited three times lower apparent H_2_ permeability than Zirfon at current densities above 300 mA cm^−2^. At 60 °C, both of the cells assembled with 3FBP‐Te and Zirfon showed higher apparent H_2_ permeability than at 40 °C mainly due to the reduced viscosity and increasing mobility of the electrolyte. Similarly, the cell assembled with 3FBP‐Te presented better hydrogen barrier properties than Zirfon, likely due to the amorphous phase within the 3FBP‐Te as indicated by the XRD results.

### 2.3 Stability Analysis

The H_2_ crossover evolution shown in Figure S8 indicates a sudden increase in H_2_ in O_2_ level after 90 h at 80 °C, which was due to membrane fracture along the edge of the sealing gasket (Figure S9a). In order to explore if the membrane fracture observed at 80 °C was caused by chemical or mechanical degradation, the 3FBP‐Te membranes were taken out, rinsed with water and recast. Photographic images of the membrane after washing and re‐casting are shown in Figure S9b. During the washing process, the membrane was found to be partly soluble in water, likely due to KOH residuals and the strong hydrophilicity of the tetrazolide ion form.

**Figure 9:**
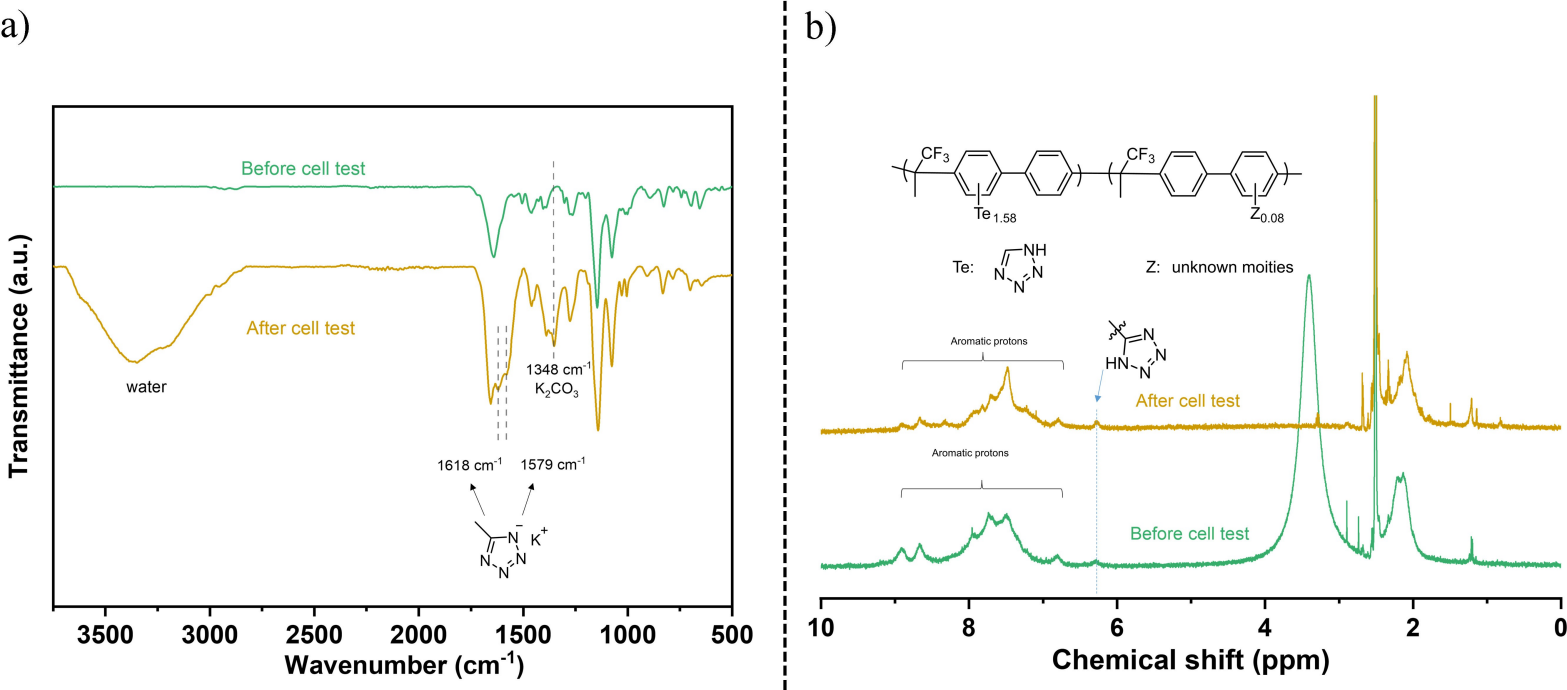
FTIR (a) and ^1^H‐NMR spectrum (b) of 3FBP‐Te membrane after 160 h electrolysis testing.

The FTIR of the 3FBP‐Te membrane after the cell test is shown in Figure 9a. It can be seen that the characteristic tetrazole absorption shifted from 1650 cm^−1^ to 1618 cm^−1^ and 1579 cm^−1^ following the deprotonation of the tetrazole moieties and cation exchange with potassium.^[59]^ Another obvious absorption band is found around 3400 cm^−1^, which is attributed to the ‐OH of the absorbed water. The absorption band around 1348 cm^−1^ is due to the potassium carbonate formed from the reaction with carbon dioxide from the atmosphere. Besides, little difference was observed from the FTIR spectra results. The ^1^H NMR spectrum, as shown in Figure 9b, was essentially identical to that of the starting materials, although a detailed interpretation is complicated due to the limited solubility of 3FBP‐Te. However, a new signal at around 8.2 ppm can be seen after cell testing, which could potentially be connected to substitution of bromide residuals to form phenolic moieties or hydrolysis of nitrile residuals to form carboxylic acid units.

The FTIR data at wavenumbers below 1200 cm^−1^ would normally provide information related with phenyl chemical structure and substitution. However, in this case, no vast difference was observed suggesting excellent chemical resilience. A plausible explanation is that the dynamic environment in the cell resulted in swelling and morphology changes that ultimately resulted in mechanical deterioration via dissolution. To test this hypothesis, the 3FBP‐Te membranes were immersed into 80 °C KOH solutions with concentrations from 5 to 40 wt.A % for over 12 h, and water was included as reference. The membrane color gradually changed from yellow to dark brown with increasing KOH concentration, while the water treated membrane retained its original color, as shown in Figure S10. Moreover, at low KOH concentrations, i. e., 5 and 10 wt %, the solution turned yellow, likely due to partial dissolution of the membrane. This clearly shows that a local KOH concentration variation could have a major impact on the physicochemical properties of the membrane.

In brief, 3FBP‐Te presented promising cell performance at 80 °C. The swelling, solubility and mechanical characteristics were found to be highly dependent on the concentration of the surrounding electrolyte, which could lead to premature mechanical failure at cell level where the specific conditions can vary depending on current setpoints, electrolyte balancing, and flow rates. Crosslinking may therefore be needed to balance the electrolyte uptake and to prevent excessive swelling under operating conditions. It is anticipated that further exploration of the degradation modes of the tetrazole functionalized poly(arylene alkylene) membranes under the operating conditions will reveal important information on how to design hydrophilic membranes based on superacid catalyzed polyhydroxyalkylation polymers with high H_2_ barrier property and long‐term durability.

## 3 Conclusions

A poly(arylene alkylene) carrying tetrazole pendants with degrees of functionalization exceeding 150 % was successfully synthesized and characterized as a new class of alkaline ion‐solvating membranes. The physicochemical properties of the membrane in terms of swelling, electrolyte uptake, mechanical strength and conductivity could be tuned by altering the concentration of the surrounding KOH electrolyte. After comprehensive evaluation of the conductivity and mechanical robustness, 30 wt. % KOH was chosen to serve as the supporting electrolyte for cell testing. The cell assembled with a 95 μm 3FBP‐Te membrane showed polarization properties comparable to that of commercial Zirfon separators, and low apparent permeability despite being several times thinner. The membranes showed excellent chemical stability under technologically relevant conditions and device performance stability is expected to be enhanced by tuning the swelling at elevated temperatures.

## Experimental Section

### Materials

1,1,1‐Trifluoroacetone (97 %), biphenyl (99 %), trifluoromethanesulfonic acid (TFSA, 98 %), trifluoroacetic acid (TFA, 98 %), trifluoroacetic anhydride (TFAA, 99 %), copper(I) cyanide (CuCN, 99 %), bromine (99 %), ammonium chloride (NH_4_Cl, 99 %), sodium azide (NaN_3_, 99 %) ethylenediaminetetraacetic acid sodium salt (EDTA‐Na_2_, 99 %), sodium hydroxide (NaOH, 97 %), dichloromethane (DCM, 99 %), 1‐methyl‐2‐pyrrolidone (NMP, 99 %), dimethylformamide (DMF, 99 %), deuterated chloroform (CDCl_3_, 99 atom % D) and deuterated dimethyl sulfoxide (DMSO‐*d*_6_, 99 atom % D) were obtained from Sigma Aldrich. Potassium hydroxide (KOH, 88 %) and methanol (99 %) were purchased from VWR Chemicals. Iron powder (99 %) was obtained from Fluka. Zirfon Perl UTP 500 was purchased from Agfa. Nickel foam (pore size 450 μm, thickness 1.6 mm) was obtained from Alantum. NMP and DMF were dried over 4 Å molecular sieves prior to use. Other chemicals were used as received.

### Synthesis and Membrane Casting

*Synthesis of poly[[1,1′‐biphenyl]‐4,4′‐diyl(1,1,1‐trifluoropropan‐2‐yl)] (3FBP)*: To a 1000 mL nitrogen purged dry three‐neck round‐bottom flask, 1,1,1‐trifluroacetone (24.6 g, 0.22 mol) and biphenyl (30.8 g, 0.20 mol) were added and dissolved in 425 mL DCM. The mixture was stirred for 30 min in an ice‐cooled bath, and TFSA (85 mL, 1.06 mol) was thereafter added slowly from an addition funnel. The reaction mixture was stirred at room temperature for 28 h. 8 mL TFAA was added and the viscous mixture was stirred for another 2 h, and thereafter poured slowly into methanol. White fibers formed instantly, which were isolated by filtration and washed thoroughly with methanol. After drying under vacuum overnight at 60 °C, 3FBP was obtained as white fibers with a yield of 50.22 g (98 %).

*Bromination*: 3FBP (20 g, 80.65 mmol) was dissolved in 500 mL DCM in a double‐neck round‐bottom flask equipped with a condenser. An aqueous NaOH trap was connected to the top of the condenser to absorb evolved acidic gas. Iron powder (5.23 g, 93.73 mmol) and bromine (30 mL, 1.17 mol) were then added to the homogenous solution. The reaction mixture was kept stirring at reflux overnight. The solution was poured into methanol, resulting in instant precipitation of gray‐white fiber of brominated 3FBP (3FBP‐Br). The precipitate was isolated by filtration, thoroughly washed with methanol and water to obtain 34 g of the 3FBP‐Br intermediate after drying. The bromination degree was estimated to 200 % based on the weight increase, which was used to define the reaction stoichiometry in the subsequent reaction steps.

*Substitution of bromine with nitrile*: To a 250 mL three‐neck round‐bottom flask equipped with a magnetic stirrer, condenser and nitrogen inlet, 3FBP‐Br (2.0 g, 4.93 mmol) was dissolved in 50 mL NMP at 80 °C. After the complete dissolution, CuCN (1.40 g, 15.56 mmol) was added, and the solution was stirred for 72 h at 166 °C. The solution was cooled to room temperature and poured into water and washed with aqueous EDTA‐Na_2_ solution to scavenge the copper ions. An amount of 1.26 g of the nitrile functionalized 3FBP (3FBP‐CN) was obtained as a brownish powder after rinsing in water and drying under vacuum at 80 °C.

*Conversion of nitriles to tetrazoles*: To a 100 mL round‐bottom flask equipped with a condenser, 3FBP‐CN (1.2 g, 4.03 mmol) was dissolved in 24 mL DMF at 80 °C. NaN_3_ (0.86 g, 13.3 mmol) and NH_4_Cl (0.70 g, 13.3 mmol) were added, and the temperature was increased to 130 °C. After keeping the reaction stirred for 48 h, the solution was poured into water, and the pH was adjusted to around 4 to precipitate the polymer. The precipitate was washed in water until neutral pH, and 1.40 g of the tetrazole functionalized 3FBP (3FBP‐Te) was obtained as a deep brownish powder after filtration and drying at 80 °C.

*Membrane casting*: 3FBP‐Te (0.6 g) was dissolved in 10 mL NMP at 80 °C under ultrasonication overnight. The obtained deep brown viscous solution was cast on cleaned Petri dishes (9 cm diameter) by solvent evaporation at 65 °C for 72 h. The obtained 3FBP‐Te membrane was delaminated from the glass substrates after immersing in water overnight, and dried under vacuum at 80 °C for another 24 h. The membranes were equilibrated in aqueous KOH at concentration of 5, 10, 20 and 30 wt. % for 24 h before use.

### Characterization

The molecular weight and polydispersity (*Đ*) of 3FBP was determined by size exclusion chromatography (SEC) in THF, using an OMNISEC from Malvern Instrument with a refractive index (RI) detector. A TGuard (Org. Guard Col 10×4.6 mm) was used as guard column, two T6000 M (General mixed Org. 300×8.0 mm) were used as analytical columns. Eight polystyrene standards (*M*_p=_3,530,000 and 184,000 g mol^−1^ from Polymer Standards Service, *M*_p=_1,184,000 g mol^−1^ from Polymer Laboratories, *M*_p=_93,800 and 9,000 g mol^−1^ from Sigma‐Aldrich, *M*_p=_35,000 g mol^−1^ from Waters and *M*_p=_17,500 and 3,000 g mol^−1^ from Polysciences Inc.) were used for calibration. 3FBP was dissolved 24 h before the measurement and was filtered through a 0.2 μm PTFE filter before the injection. The column temperature was 35 °C and the flow rate was 1 mL min^−1^. The inherent viscosity (*η*_inh_) was determined by using an Ubbelohde suspended level viscometer with polymer concentration 0.5 g dL^−1^ in NMP at 21 °C. Fourier transform infrared (FTIR) spectra were recorded using a PerkinElmer Spectrum TWO equipped with an attenuated total reflectance (ATR) accessory in the IR frequency range from 4000 to 500 cm^−1^. ^1^H NMR spectra were recorded using a Bruker Avance 400 MHz or spectrometer, using deuterated dimethyl sulfoxide (DMSO‐*d*_6_) and chloroform (CDCl_3_) as solvent. X‐ray photoelectron spectroscopy (XPS) was conducted with an ESCALAB 250Xi and a monochromatized Al Kα X‐ray source. Scofield relative sensitivity factors were used for quantification. X‐ray diffraction (XRD) was carried on an Aeris powder diffractometer for 2*θ* angles between 5° and 50°. Thermogravimetric analysis (TGA) was carried out using a TA Instruments TGA Q500. The samples were first dried at 60 °C under vacuum overnight. Prior to analysis, the samples were kept at 120 °C during 20 min in the instrument to evaporate traces of water. The measurements were then performed under nitrogen atmosphere from 50 to 600 °C at a heating rate of 10 °C min^−1^. Scanning electron microscopy (SEM) was carried out using a Zeiss EVO MA10. The cross sections of the membranes were fabricated by cryogenic fracturing in liquid nitrogen, followed by sputter coating with gold. Stress‐strain curves were recorded using a Mecmesin MultiTest‐dV Low‐force materials tester at a crosshead speed of 20 mm min^−1^, using 5 mm wide specimens. Before electrolyte uptake and swelling measurement, all the membranes were dried under vacuum at 60 °C overnight. After recording the dry weight, the membranes were transferred to different concentration KOH solutions varying from 5 to 30 wt. % and kept at room temperature for 24 h. The electrolyte uptake, *EU* (i. e. the sum contributions from water and KOH), was calculated according to Equation 1, where *W*_w_ and *W*_d_ is the weight of the wet and dry membrane, respectively.

(1) $$EU=\frac{W_{w}-W_{d}}{W_{d}}\times 100\;\%$$

The thickness swelling (swelling ratio, *SR*) was measured by immersing the samples in the electrolytes of different concentration KOH solutions at room temperature for 24 h, in parallel with the electrolyte uptake test, and calculated according to Equation 2, where *T*_w_ and *T*_d_ is the thickness of the wet and dry membranes, respectively:

(2) $$SR=\frac{T_{w}-T_{d}}{T_{d}}\times 100\;\%$$

### Ion Conductivity and Electrolysis Testing

The conductivity measurements and electrolysis tests were performed as described elsewhere,^[20]^ by sandwiching the membrane between two Ni foam electrodes in a two‐compartment PTFE cell with aqueous KOH in the cell chambers. The through‐plane resistance was determined by electrochemical impedance spectroscopy (EIS), using a Gamry Reference 3000 with an AC perturbation of 5.0 mV amplitude in the frequency range from 1000 Hz to 100 kHz. The resistance was taken as the real component of the impedance *Z*_re_ at 0° phase angle, and the conductivity *σ* was calculated according to Equation 3, where *d* and *S* are the thickness of the membrane and geometric surface area of the Ni electrodes, respectively. The membrane samples were equilibrated in KOH solutions of different concentrations for at least 24 h before the measurement.

(3) $$\sigma =\frac{d}{Z_{re}\times S}$$

Single cell electrolysis tests were conducted by sandwiching the pre‐equilibrated membrane between two pieces of uncatalyzed Ni‐foam electrodes (compressed to 300 μm) with an active geometric area of 10 cm^2^. Flat sheet polytetrafluoroethylene (PTFE) gaskets with suitable thickness were applied for sealing. The thickness of the gaskets was adjusted to match the thickness of the membrane and electrodes to avoid mechanical damage of the membrane due to compression. During the test, two heating rods and a thermocouple were inserted into the end plates to control the temperature, and 30 wt. % KOH (aq.) was circulated on both sides at a flow of 80 mL min^−1^ by two separated gear pumps, in a partially separated electrolyte flow mode. In order to stabilize the cell, a break‐in current density of 100 mA cm^−2^ was applied at 40 °C for 1 h. The polarization testing was then followed in sequence with the temperature of 40, 60 and 80 °C. The H_2_ content in the outlet from the anode compartment was recorded with a hydrogen sensor from Geopal Systems (after drying through a silica gel column). The H_2_ crossover was determined at current densities of 50, 100, 150, 200 and 300 or 400 mA cm^−2^ in sequence at 40, 60 and 80 °C during continuous N_2_ flush of 56 mL min^−1^. Each current density was maintained for 4 h and the N_2_ purging, denoted as $N_{N_{2}}$ , was applied to alleviate the H_2_ sensor latency and optimize the signal consistency. In other words, the measured H_2_ level in the gas sensor is the molar fraction of H_2_ in the mixture of H_2_, O_2_ and the introduced N_2_, which is denoted as $X_{H_{2}}$ . The molar fraction of O_2_ and N_2_ are denoted $X_{O_{2}}$ and $X_{N_{2}}$ respectively. The rate of O_2_ formation $N_{O_{2}}$ , was calculated based on the current setpoint, assuming 100 % faradaic efficiency for O_2_ evolution, assuming negligible diffusive losses across the membrane. The H_2_ crossover flux $\varphi_{H_{2}}$ is equivalent to the rate of H_2_ transport into the anodic side $N_{H_{2}}$ and can be written as:

(4) $$\varphi_{H_{2}}=N_{H_{2}}=\frac{N_{O_{2}}+N_{N_{2}}}{1-X_{H_{2}}}X_{H_{2}}$$

From the H_2_ flux, the relative H_2_ in O_2_ level $\theta_{H_{2}}$ (HTO) can be calculated according to Equation 5:

(5) $$\theta_{H_{2}}=\frac{\varphi_{H_{2}}}{{N_{O_{2}}+\varphi }_{H_{2}}}$$

According to Fick′s law and Henry′s law, the apparent permeability can be calculated as shown in Equation 6.[^20^, ^24^, ^25^]

(6) $$\epsilon_{H_{2}}=\varphi_{H_{2}}\frac{d}{\Delta p_{H_{2}}}$$

The partial pressure of H_2_ in anode is negligible compared with cathode side. The H_2_ production in cathode is under atmosphere pressure, thus $\Delta p_{H_{2}}$ is assumed to be 1 atm.

## Supporting Information

Supporting Information is available from the Wiley Online Library or from the author.

## Conflict of Interests

The authors declare no conflict of interest.

4

## Supporting information

As a service to our authors and readers, this journal provides supporting information supplied by the authors. Such materials are peer reviewed and may be re‐organized for online delivery, but are not copy‐edited or typeset. Technical support issues arising from supporting information (other than missing files) should be addressed to the authors.

Supporting Information

## Data Availability

The data that support the findings of this study are available from the corresponding author upon reasonable request.
