# Supplementary material for: Poly(Arylene Alkylene)s with Tetrazole Pendants for Alkaline Ion‐Solvating Polymer Electrolytes
Source: ChemSusChem. 2024 Aug 8;17(23):e202400844. doi: 10.1002/cssc.202400844 (PMC11632586; doi:10.1002/cssc.202400844)
Supplement: Supplementary file 1 — Supporting Information [file CSSC-17-e202400844-s001.pdf]

ChemSusChem

Supporting Information

Poly(Arylene Alkylene)s with Tetrazole Pendants for Alkaline Ion-Solvating Polymer Electrolytes

Yifan Xia, Sinu C. Rajappan, Si Chen, Mikkel Rykær Kraglund, Dmytro Serhiichuk, Dong Pan, Jens Oluf Jensen, Patric Jannasch, and David Aili*

Supporting Information

Poly(arylene alkylene)s with Tetrazole Pendants for Alkaline Ion-Solvating Polymer Electrolytes

*Yifan Xia, Sinu C. Rajappan, Si Chen, Mikkel Rykær Kraglund, Dmytro Serhiichuk, Dong Pan, Jens Oluf Jensen, Patric Jannasch and David Aili**

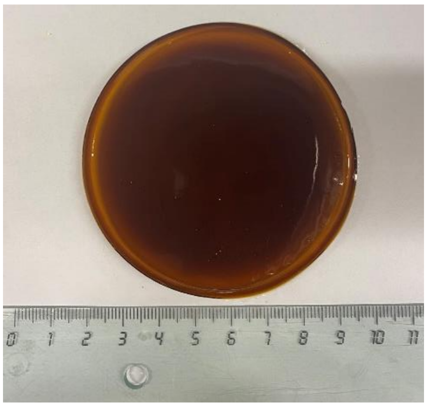

Figure S1. Photograph of 3FBP-Te membrane after solution casting.

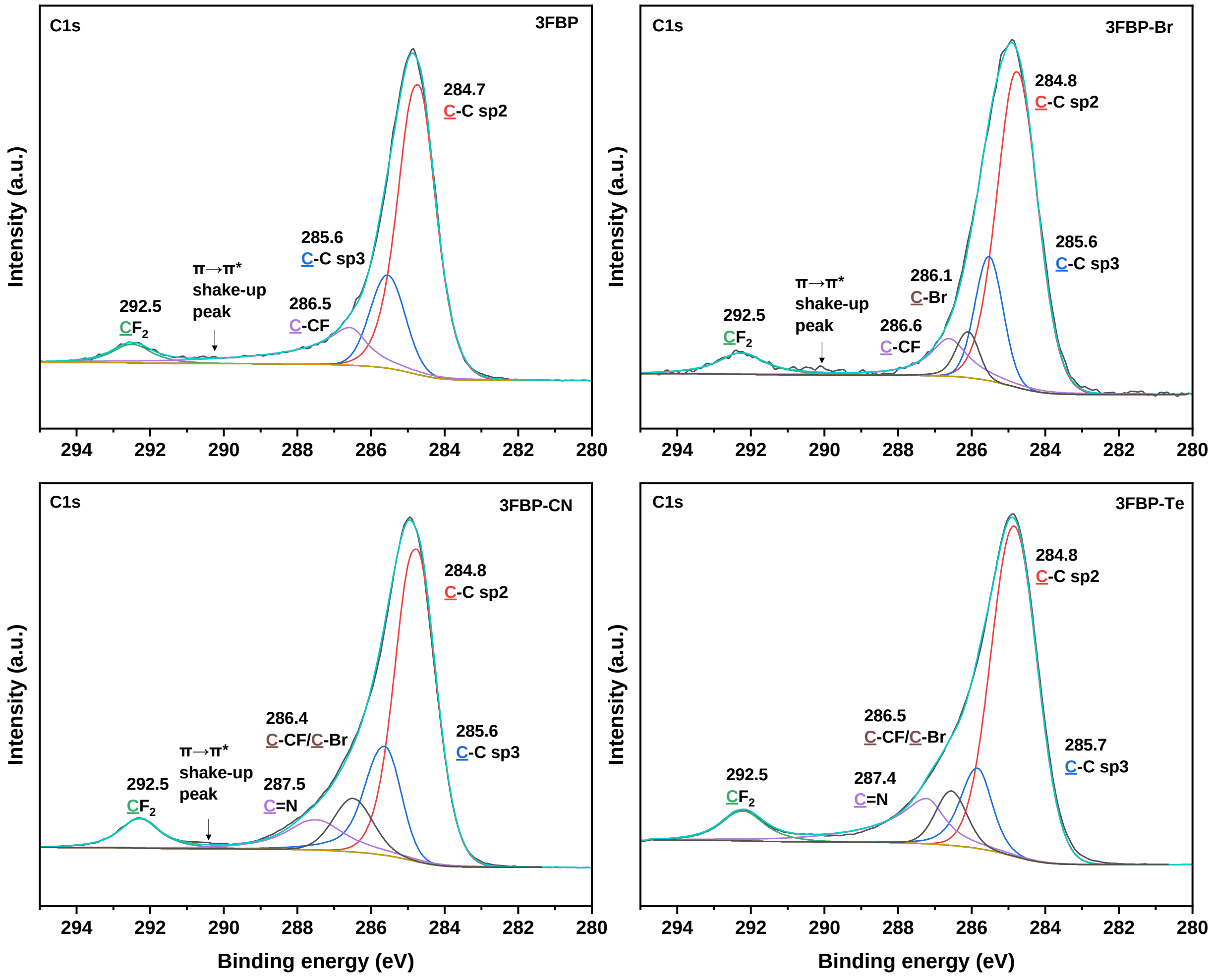

Figure S2. Deconvolution of XPS spectra of C1s from 3FBP, 3FBP-Br, 3FBP-CN and 3FBP-Te.

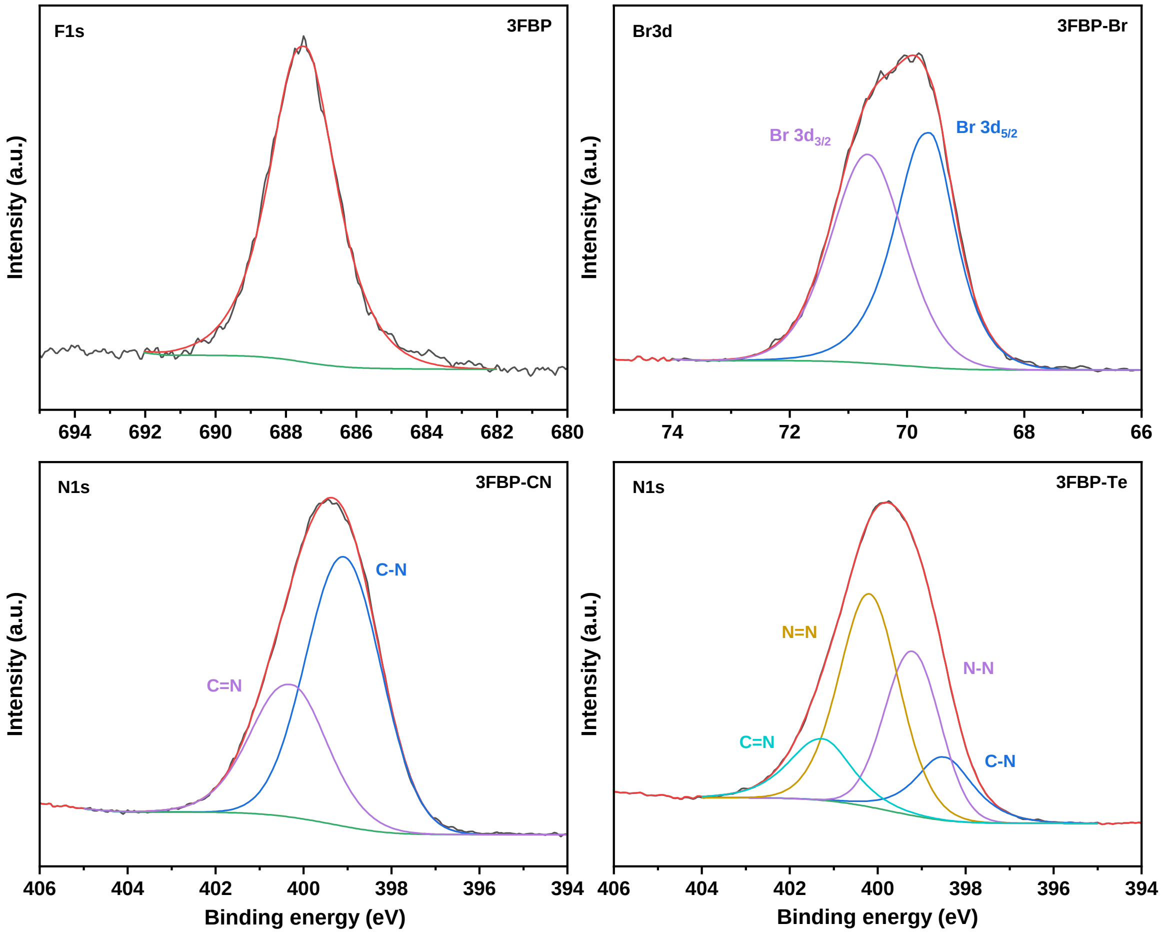

Figure S3. Deconvolution of XPS spectra of F1s and Br 3d for 3FBP and 3FBP-Br, respectively, and N 1s for 3FBP-CN and 3FBP-Te.

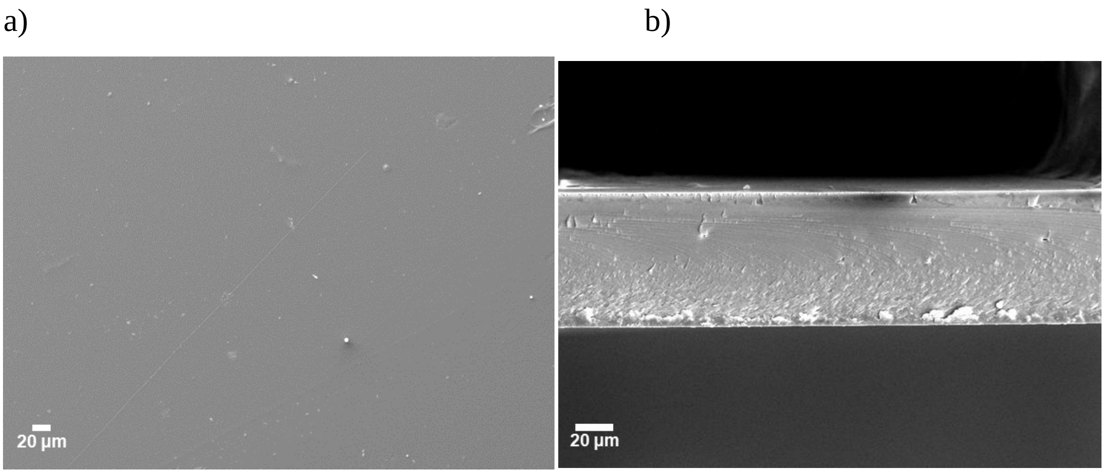

Figure S4. SEM images of the surface (a) and cross-section (b) of the 3FBP-Te membrane.

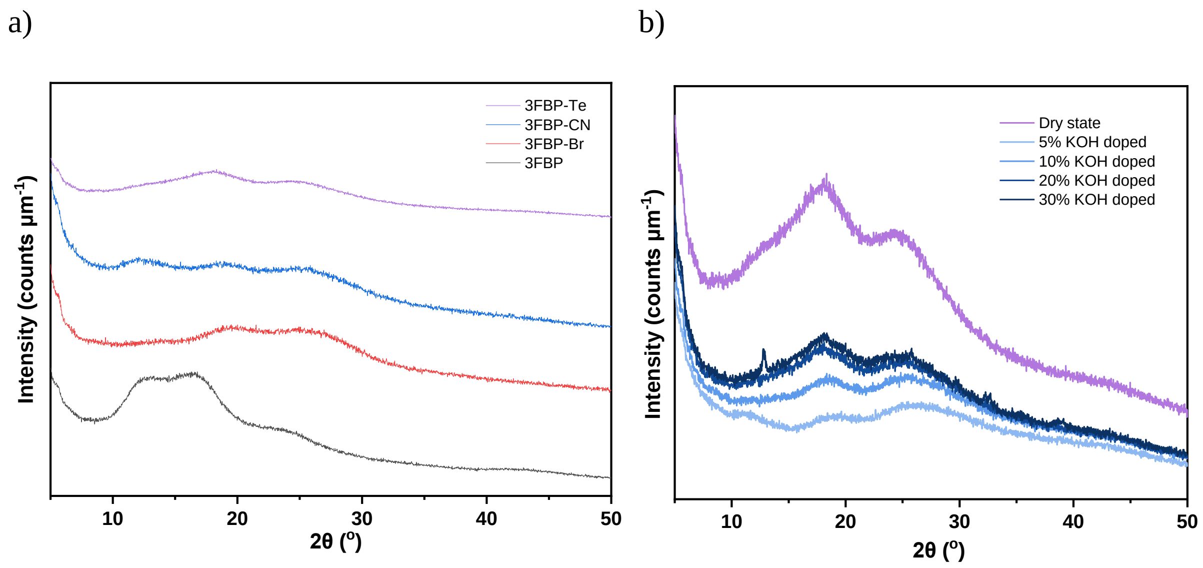

Figure S5. XRD data of 3FBP, 3FBP-Br, 3FBP-CN and 3FBP-Te membranes (a), and of 3FBP-Te after equilibration in aqueous KOH with concentrations of 0-30 wt.% (b).

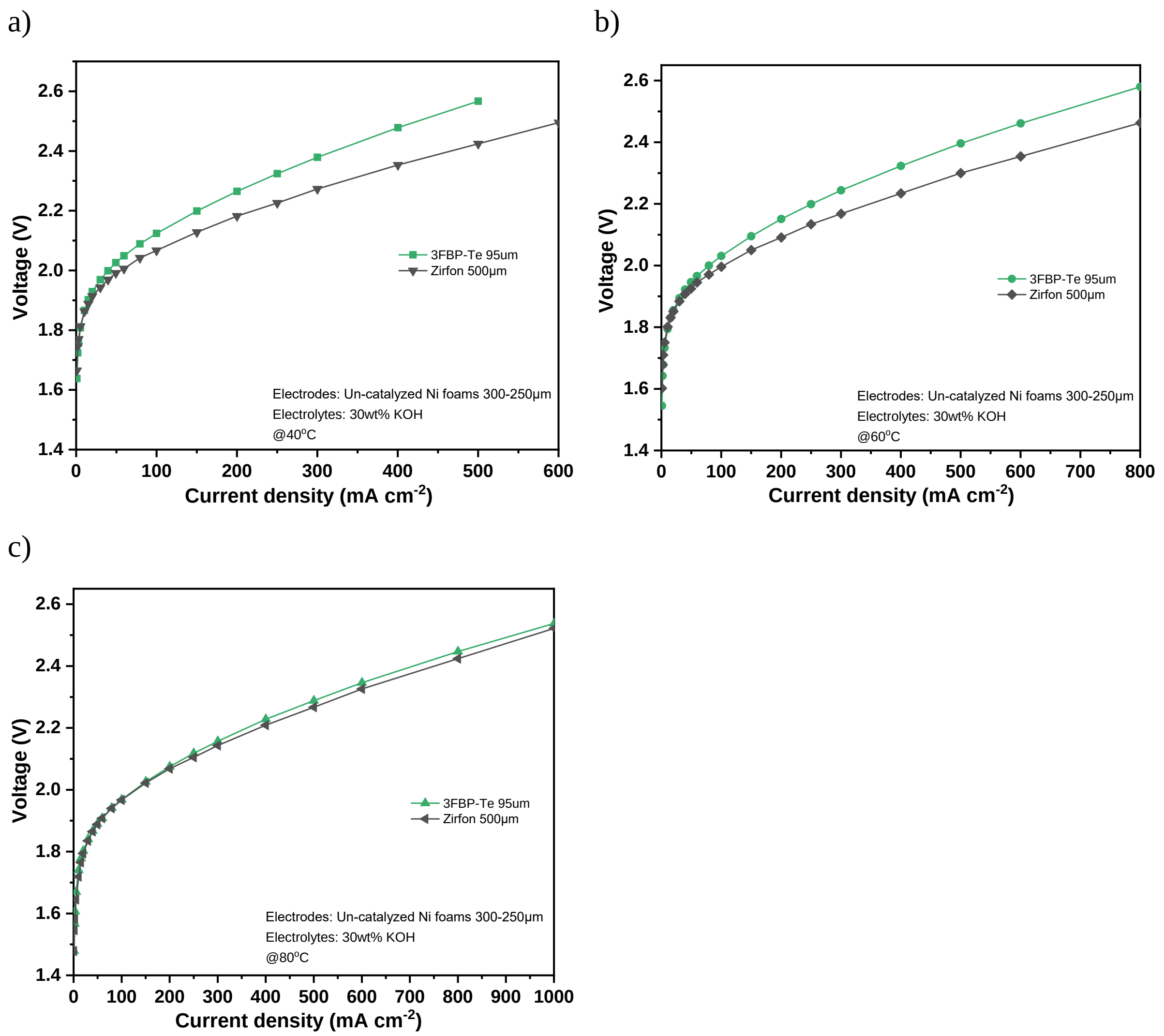

Figure S6. Water electrolysis polarization curves for cells equipped with 3FBP-Te and Zirfon in 30 wt% tested at 40 °C (a), 60 °C (b) and 80 °C (c).

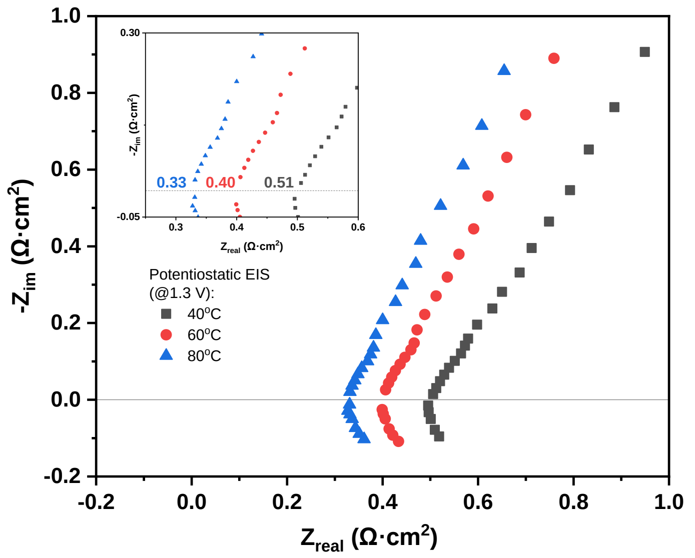

Figure S7. The potenstiostatic EIS of the cell equipped with 3FBP-Te at 40-80 °C.

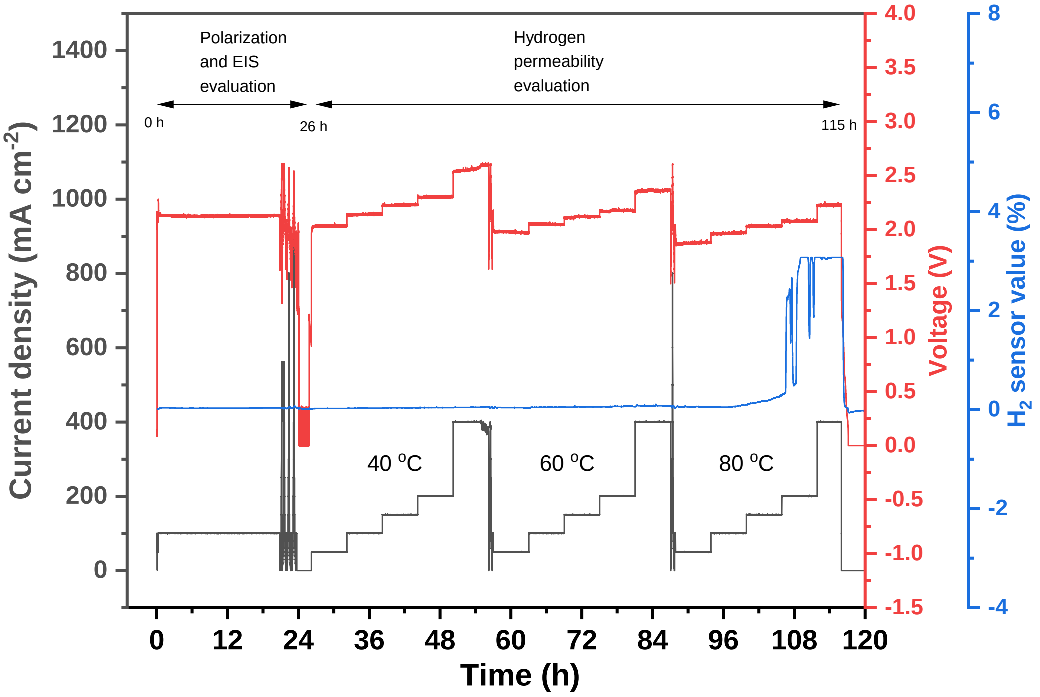

Figure S8. Voltage evolution and raw H_2 level (not corrected for N_2 fraction) as function of time for the cell assembled with 3FBP-Te.

a) b)

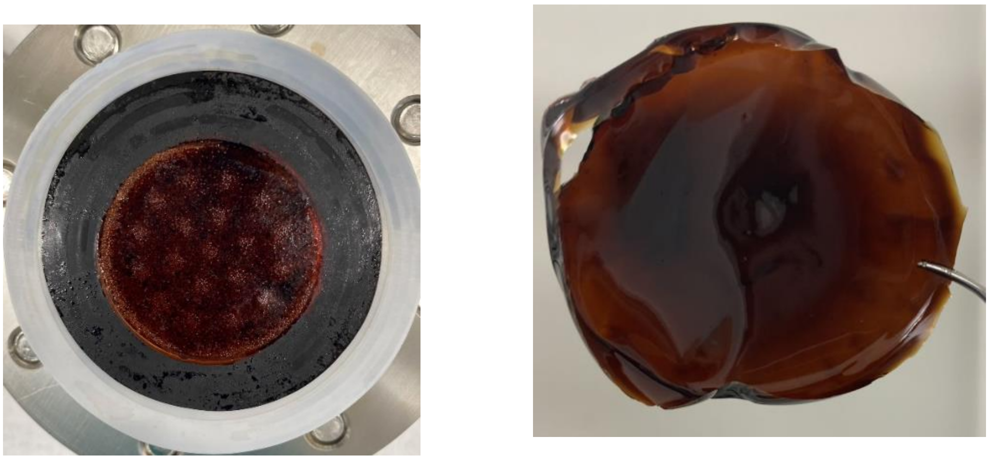

Figure S9. Photograph of 3FBP-Te membrane after 115 h electrolysis tests cell test (a) and after washing and re-casting (b).

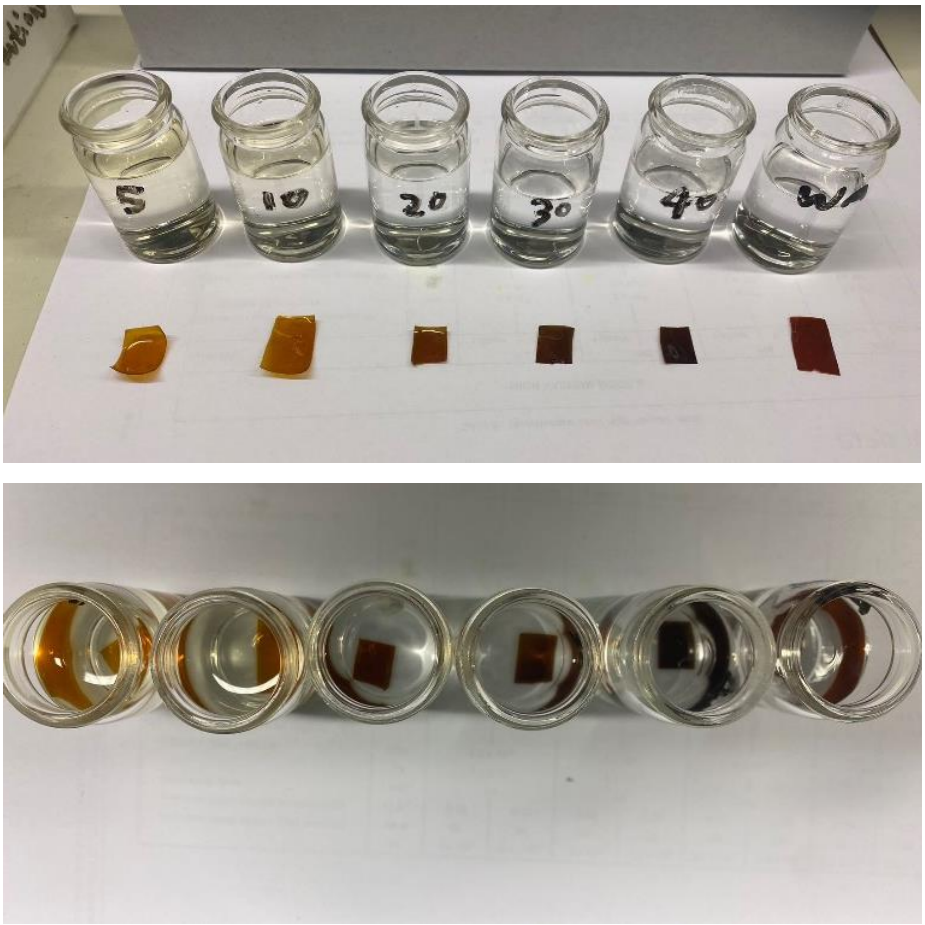

Figure S10. Photograph of 3FBP-Te membranes immersed in different KOH concentration solutions over 12 h.
